# MiR-130a-3p regulates FUNDC1-mediated mitophagy by targeting GJA1 in myocardial ischemia/reperfusion injury

**DOI:** 10.1038/s41420-023-01372-7

**Published:** 2023-02-25

**Authors:** Yan Yan, Liu-yang Tian, Qian Jia, Yang Han, Yu Tian, Hui-ning Chen, Sai-jia Cui, Jie Xi, Yong-ming Yao, Xiao-jing Zhao

**Affiliations:** 1 Medical Innovation Research Division, the Chinese PLA General Hospital, Beijing, 100853 People’s Republic of China; 2 School of Medicine, Nankai University, Tianjin, 300071 People’s Republic of China

**Keywords:** Mitophagy, Mitophagy

## Abstract

Understanding the complex pathogenesis in myocardial ischemia/reperfusion (I/R) injury (IRI) is an urgent problem in clinical trials. Increasing pieces of evidence have suggested that miRNAs are involved in the occurrence and development of heart diseases by regulating mitochondria-related gene expression. Mitochondria have been acknowledged as the key triggers of cardiac I/R injury. However, the potential impact of miR-130a on mitochondria remains unclear in myocardial IRI. Exploring the regulatory mechanism of miR-130a on mitochondria may provide a new target for IRI therapy. In the present study, we found that miR-130a significantly increased in acute myocardial infarction (AMI) patients and myocardial I/R rats. MiR-130a could downregulate the viability of cardiomyocytes and the knockdown of miR-130a could protect the viability of cardiomyocytes under hypoxia-reoxygenation (HR). Over-expression of miR-130a resulted in mitochondrial dysfunction. It was evidenced by decreases in mitochondrial ATP production, mitochondrial membrane potential (MMP), and an increase in reactive oxygen species (ROS) production. However, suppression of miR-130a could protect against mitochondrial damage, show elevation of mitochondrial ATP production rate and MMP, and reduce ROS production. We further explored the effect of miR-130a on the mitochondrial quality control (QMC) system by determining mitochondrial-protein-specific proteases and analyzed mitochondrial morphology by fluorescence imaging and electron microscopy, respectively. It was noted that miR-130a could suppress mitochondrial fusion and FUNDC1-mediated mitophagy to accelerate myocardial IRI. Moreover, we investigated the potential miR-130a targeted mitochondria-related genes to understand the regulatory mechanism of miR-130a in the setting of myocardial IRI. It was revealed that miR-130a targeted GJA1, and GJA1 rescued IRI by enhancing ATP production rate and oxidative phosphorylation, meanwhile protecting cell viability, MMP, and activating mitophagy. In addition, the knockdown of miR-130a significantly activated FUNDC1-mediated mitophagy, while the knockdown of GJA1 reversed the relevant response. Collectively, our findings suggest that miR-130a regulates FUNDC1-mediated mitophagy by targeting GJA1 in myocardial IRI.

## Introduction

Myocardial Ischemia/reperfusion (I/R) injury is a common and serious pathophysiological phenomenon during cardiac surgery, heart transplant, MI, and cardiac arrest [1]. Due to its complex pathogenesis, there is still no effective method to treat myocardial I/R injury (IRI) in clinical practice [2–5]. Understanding the complex pathogenesis of myocardial IRI is an urgent problem solved in clinical trials [1]. MiRNAs and mitochondria are critical to involve in cardiac pathophysiology [6]. miRNAs play an important role in the pathogenesis and prognosis of cardiac diseases, and can also be an important therapeutic target [7]. MiRNAs are a class of short and highly conserved non-coding RNAs [8]. Increasing pieces of evidence have suggested that miRNAs are involved in the occurrence and development of heart disease by regulating the mitochondria-related genes, including biogenesis, fission, and mitophagy genes [9, 10]. MiR-130a is a high-expression gene in the heart [11, 12]. Current studies have reported that miR-130a is an important regulator involved in various cardiovascular diseases [11–15]. However, the impact of miR-130a on mitochondrial function remains unknown. Mitochondria have been acknowledged as the key triggers of cardiac IRI [16]. Mitochondria act as “energy factories”, providing energy to all cells [17]. As the most metabolically active organ, the heart needs a lot of mitochondria to provide energy for keeping systolic and diastolic functions. Mitochondrial dysfunction is associated with cardiac disease development and is caused by reduced ATP synthesis, excessive reactive oxygen species (ROS) production, the disorder of mitochondrial dynamics, and the abnormality of mitophagy [18]. Emerging studies have suggested that mitochondrial homeostasis plays an important role in mitochondrial dysfunction, and mitophagy is a crucial process in regulating mitochondrial homeostasis [7]. Exploring the regulation mechanism of miR-130a on mitochondria may provide a new target for myocardial IRI therapy.

MiRNAs regulate gene expression by inhibiting translation or inducing mRNA degradation [19]. The genes targeted by miR-130a were predicted by the miRBase database, and GJA1 was chosen as the target for our study. Protein GJA1/CX43 is a major gap junction component in many cardiac biologic processes. Most of the previous studies on GJA1 focused on its role in information exchange and material transport between cells by forming gap junctions and semi-channels. However, studies have clarified GJA1 can regulate autophagy [20, 21] and hold mitochondrial protection in recent years [22, 23]. GJA1 is a high-expression protein in the mitochondria of cardiomyocytes [24], and some studies have indicated that GJA1 is involved in the pathogenesis of myocardial IRI [25, 26]. Nevertheless, the regulatory mechanism of GJA1 in myocardial IRI remains unclear.

In this study, we found miR-130a significantly increased in both acute myocardial infarction (AMI) patients and myocardial I/R rats. Mitochondria functional studies suggested that Overexpression of miR-130a could cause mitochondrial dysfunction, and suppression of miR-130a could protect mitochondria. Moreover, miR-130a suppressed mitochondrial fusion and FUNDC1-mediated mitophagy to accelerate myocardial HRI. Next, we found miR-130a targeted GJA1. And, GJA1 rescued IRI by enhancing ATP production rate and oxidative phosphorylation, meanwhile protecting cell viability, mitochondrial membrane potential, and activated mitophagy. At last, we confirmed knockdown of miR-130a could activate FUNDC1-mediated mitophagy, however, the knockdown of GJA1 could reverse the effect. Our findings demonstrated miR-130a regulated FUNDC1-mediated mitophagy by targeting GJA1 in myocardial IRI.

## Results

### MiR-130a increased in AMI patients and I/R rats

**Fig. 1:**
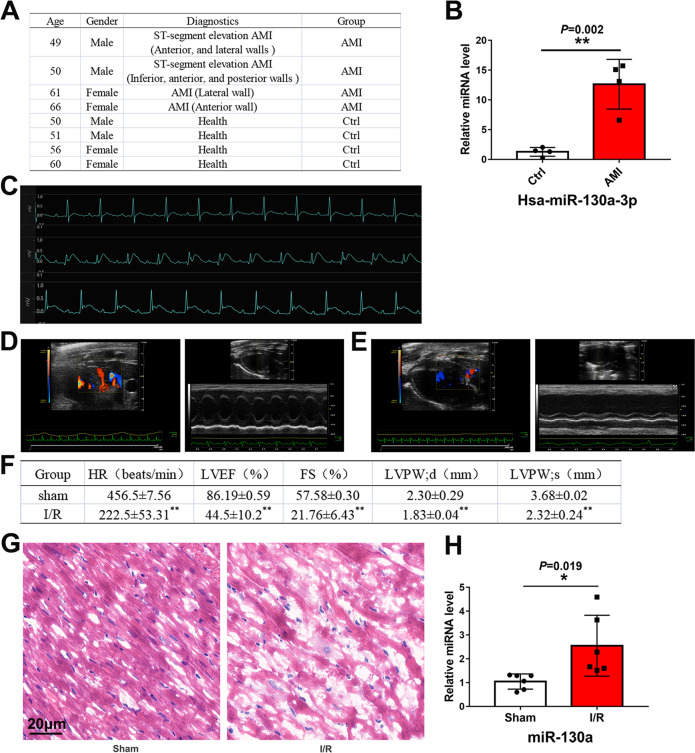
MiR-130a was increased in the blood of AMI patients and myocardial tissue of I/R rats. **A** Characteristics of the AMI patients and healthy people in our study. **B** The miR-130a-3p expression level of the AMI patients was assessed by qRT-PCR. *n* = 4 in the Ctrl group, *n* = 4 in the AMI group. **C** The continuously monitored electrocardiogram of I/R rats by electrocardiogram monitor during the period of pre-operation, ischemia, and reperfusion. **D** Cardiac ultrasonic images of the sham group. **E** Cardiac ultrasonic images of the I/R group. **F** Cardiac parameters of rats in the sham and I/R groups including HR, LVEF, FS, and LVPW; s. *n* = 6 in each group. **G** HE staining of rat myocardium in sham and I/R groups. *n* = 6 in each group. Bar = 20 μm. **H** The levels of miR-130a expression in the sham and I/R groups were assessed by qRT-PCR. U6 was used as a reference gene. *n* = 6 in each group. Data were expressed as mean ± SD; ^*^P < 0.05, ^**^P < 0.01 by two-tailed Student’s t-test. AMI acute myocardial infarction, HR hypoxia-reoxygenation, LVEF left ventricular ejection fraction, FS fractional shortening, LVPW, s left ventricular posterior wall; systolic.

To explore the role of miR-130a on myocardial IRI, we tested the miR-130a-3p expression in the peripheral blood of AMI patients treated with reperfusion. Characteristics of patients were shown in Fig. 1A. The level of miR-130a significantly increased in the AMI patients compared with the healthy person, as indicated in Fig. 1B. Next, the models of myocardial I/R rats were constructed, and Standard II lead electrocardiogram was observed during the experiment. As shown in Fig. 1C, the ST segment was elevated after myocardial ischemia and recovered after reperfusion. To investigate whether did cardiac function change in the I/R rats, cardiac parameters were measured by small animal ultrasound machines (FUJIFILM, Bothell, WA). Compared with the Sham group, HR, LVEF, FS, and LVPW; s significantly decreased in the I/R group (Fig. 1D-F). In addition, the heart histology and morphology were observed with HE staining. As shown in Fig. 1G, myocardial fibers were broken, and cardiomyocytes revealed slighter degeneration and necrosis in the I/R group. To further confirm the result that miR-130a increased in myocardial IRI, we performed an RT-qPCR test in the myocardial tissue of I/R rats and found the miR-130a expression in the I/R group was higher than that in the sham group (Fig. 1H). The above data indicated that miR-130a increased in myocardial IRI, and might play a vital role.

### MiR-130a affected the viability of HR Cardiomyocytes

**Fig. 2:**
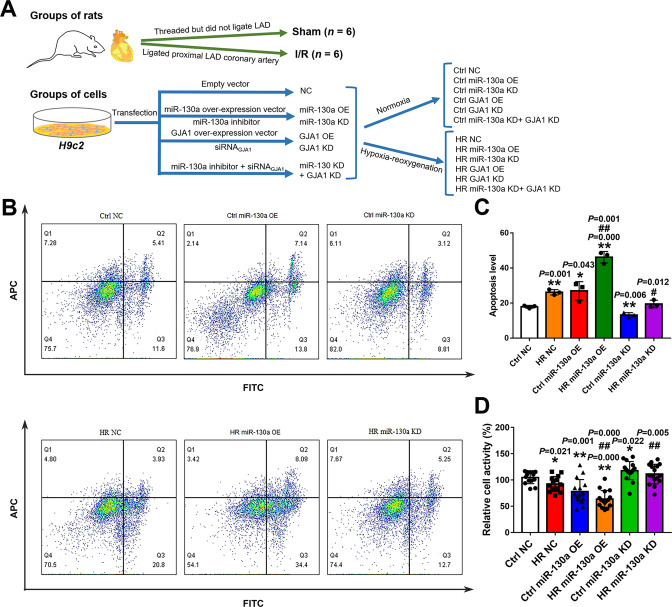
The experimental grouping of animals and cells and the effect of miR-130a on activity and apoptosis of myocardial cells. **A** We grouped the SD rats (male, 10 weeks, *n* = 12) and *H9c2* cells, and showed the treating processes. The proximal left anterior descending (LAD) coronary artery of rats (*n* = 6) was ligated to induce myocardial ischemia for 45 min followed by reperfusion for 6 h in the I/R group, and the heart samples were obtained for experiments. The sham group’s SD rats (*n* = 6) underwent the same procedures but did not ligate LAD after threading. In groups of cells, to edit the gene expression, *H9c2* cells were transfected with vectors, siRNA, or inhibitors. Next, to Simulate IRI in vitro, *H9c2* cells were grown in Hanks’ balanced salt solution under 95% N_2_ and 5% CO_2_ for 45 min, then under 20% O_2_ and 5% CO_2_ while the medium was changed to DMEM re-oxygenating for 6 h in the Hypoxia-reoxygenation (HR) groups. *H9c2* cells of the normoxia group were grown in DMEM under 20% O_2_ and 5% CO_2_. Heart tissue of rats and *H9c2* cells of each group were collected for subsequent experiments. NC: negative control; OE: over-expression; KD: knockdown. Normoxia: cells grow under 20% O_2_ and 5% CO_2_. Hypoxia-reoxygenation: cells grow in Hanks’ balanced salt solution under 95% N_2_ and 5% CO_2_ for 45 min, then under 20% O_2_ and 5% CO_2_ in DMEM for 6 h. **B**, **C** Apoptosis in *H9c2* cells was measured by Flow Cytometry under normoxia or HR conditions with miR-130a OE or miR-130a KD. *n* = 3 in each group. **D** Cell viability was determined by using a cell counting kit-8 under normoxia or HR conditions with miR-130a OE or miR-130a KD and absorbance was measured at 450 nm. *n* = 14 in the Ctrl NC and HR NC group; *n* = 16 in the Ctrl miR-130a OE, HR miR-130a OE, Ctrl miR-130a KD, and HR miR-130a KD group. Relative cell activity (%) = [(As-Ab)/(Ac-Ab)] ×100%. As: absorbance of the experimental group; Ab: absorbance of the blank group; Ac: absorbance of the Ctrl NC group. Data were expressed as mean ± SD by two-tailed Student’s t-test, ^*^P < 0.05, ^**^P < 0.01 vs. Ctrl NC; ^*#*^*P* < 0.05, ^*##*^*P* < 0.01 *vs*. HR NC. APC allophycocyanin, FITC Fluorescein Isothiocyanate.

In the following, to explore the role of miR-130a on the viability of cardiomyocytes, we tested the activity and apoptosis of *H9c2* cells under normoxia or HR conditions. As shown in Fig. 2, the over-expression of miR-130a could decrease the relative cell activity (Fig. 2D) and increase the apoptosis of *H9c2* cells (Fig. 2B, C) under normoxia and HR conditions. Besides, suppression of miR-130a could increase the relative cell activity (Fig. 2D) and decrease the apoptosis of *H9c2* cells (Fig. 2B, C) under normoxia and HR conditions. These results indicated that miR-130a could downregulate the viability of cardiomyocytes and the knockdown of miR-130a could protect the viability of cardiomyocytes.

### MiR-130a affected mitochondrial function by regulating ATP production rate, oxidative phosphorylation, reactive oxygen species content, and mitochondrial membrane potential

**Fig. 3:**
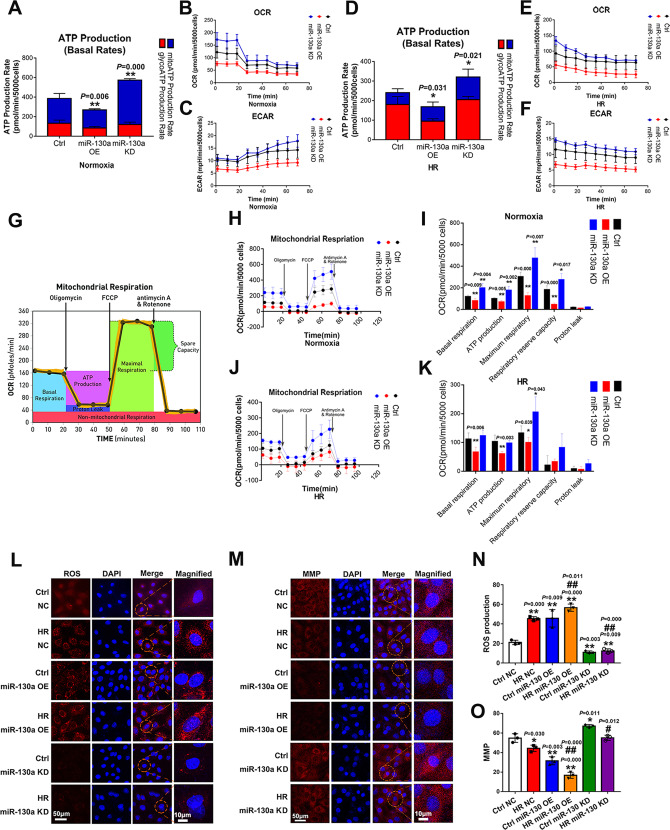
The effect of miR-130a on ATP production rate, oxidative phosphorylation, reactive oxygen species content, and mitochondrial membrane potential. **A**–**F** Total ATP production rates were measured by Agilent Seahorse analyzer under normoxia or HR conditions. The ECAR and OCR were measured under basal conditions. By obtaining these data under basal conditions and after serial addition of Rotenone/antimycin A, total cellular ATP Production Rates could be measured in real-time. *n* = 5 in each group. Data were expressed as mean ± SD; Statistical significances by two-tailed Student’s t-test, ^*^P < 0.05, ^**^P < 0.01 vs. Ctrl. The results of seahorse tests were normalized to the value per 5000 cells. **G**-**K** Mitochondrial respiration was measured including ATP production, maximal respiration, Proton leak, spare respiratory capacity, and basal respiration by Agilent Seahorse analyzer under normoxia or HR conditions. *n* = 5 in each group. Data were expressed as mean ± SD; Statistical significances by two-tailed Student’s t-test, ^*^P < 0.05, ^**^P < 0.01 vs. Ctrl. The results of seahorse tests were normalized to the value per 5000 cells. FCCP: Carbonyl cyanide-4 (trifluoromethoxy) phenylhydrazone. **L**-**O** ROS content and MMP of NC, miR-130 OE, and miR-130 KD cells under normoxia or HR condition. MMP of NC, miR-130 OE, and miR-130 KD cells under normoxia or HR condition. TMRE fluorescent dye was used to detect MMP. Nuclei were stained with DAPI. The cells were observed by laser confocal microscopy. Fluorescence intensity was measured by ImageJ software. Scale bars, 50 μm and 10 μm in magnified. *n* = 3 in each group. Data were expressed as mean ± SD; Statistical significance by two-tailed Student’s t-test, ^*^P < 0.05, ^**^P < 0.01 *vs*. Ctrl NC; ^*#*^*P* < 0.05^, *##*^*P* < 0.01 *vs*. HR NC^.^ ROS reactive oxygen species, MMP^*:*^ mitochondrial membrane potential.

To further confirm whether miR-130a played a vital role in myocardial IRI by targeting mitochondria, we examined ATP production rate, mitochondrial respiration, ROS content, and MMP in miR-130a over-expression (OE) and knockdown (KD) *H9c2* cells during hypoxia-reoxygenation (HR). As shown in Fig. 3, the Over-expression of miR-130a could cause mitochondrial dysfunction in both the control and HR group, according to the decrease of ATP production rate (Fig. 3A-F), mitochondrial respiration (Fig. 3G-K), MMP (Fig. 3M & O), and the increase of ROS production (Fig. 3L & N). Besides, suppression of miR-130a could protect mitochondria in both the Ctrl (normoxia) and HR group, according to the increase of mitochondrial ATP production rate (Fig. 3A-F), mitochondrial respiration (Fig. 3G-K), MMP (Fig. 3M & O), and the decrease of ROS production (Fig. 3L & N). Taken together, these results indicated that the increase of miR-130a expression aggravated mitochondrial dysfunction in myocardial hypoxia-reoxygenation injury (HRI).

### MiR-130a exerted a mitochondrial quality control system by mediating mitochondrial dynamics and mitophagy

**Fig. 4:**
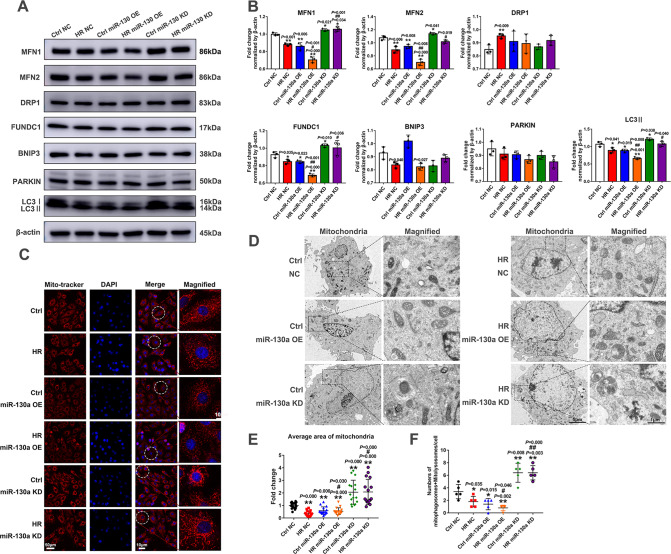
The impacts of miR-130a on mitochondrial dynamics and mitophagy. **A**, **B**. Western blot was used to detect MFN1, MFN2, DRP1, FUNDC1, BNIP3, PARKIN, and LC3II expression of miR-130a OE and miR-130a KD cells under Ctrl or HR. *n* = 3 in each group. **C**. Mitochondrial morphology by fluorescence imaging of miR-130a OE and miR-130a KD cells under Ctrl or HR. Mitochondria were stained with Mito-tracker (red), and nuclei were stained with DAPI. Scale bars, 50 μm and 10 μm in magnified. **E** The fold change in the average area of mitochondria with miR-130a OE or miR-130a KD. *n* = 16 mitochondria from three independent experiments. **D** Representative electron microscopy images from miR-130a OE and miR-130a KD cells under Ctrl or HR. Scale bars, 5 μm and 1 μm in magnified. **F** Numbers of mitophagosomes + mitolysosomes/cell with miR-130a OE or miR-130a KD. *n* = 5 cells from three independent experiments. Data were expressed as mean ± SD; Statistical significance by two-tailed Student’s t-test, ^*^P < 0.05, ^**^P < 0.01 *vs*. Ctrl; ^*#*^*P* < 0.05, ^*##*^*P* < 0.01 *vs*. HR NC.

The next question was, after mitochondrial dysfunction in myocardial IRI, how did the mechanisms responsible for the elimination of dysfunctional mitochondria work? Studies have shown mitochondrial quality control (QMC) system including mitochondrial dynamics and mitophagy, is essential for mitochondrial function following injury [27]. Therefore, we explored whether miR-130a could further affect mitochondrial dynamics and mitophagy in myocardial IRI. There are some mitochondrial-protein-specific proteases in the QMC as follows: Mitofusins 1 and 2 (MFN1, MFN2) involve in the fusion, the GTP-dependent dynamin-related protein 1 (DRP1) regulates the fission, and Bcl2/adenovirus E1B 19 kDa protein-interacting protein 3 (BNIP3), FUN14 domain containing 1 (FUNDC1), PARKIN, microtubule-associated protein 1 A/1B-light chain 3 (LC3) cause mitophagy. Western blot (WB) results showed MFN1, MFN2, FUNDC1, and LC3II expression significantly decreased in the HR NC group, compared with the Ctrl NC group. Similarly, miR-130a over-expression caused MFN1, MFN2, FUNDC1, and LC3II expression to decrease under normoxia and HR conditions. Meanwhile, MFN1, FUNDC1, PINK1, and LC3II expression significantly increased in the miR-130a KD group, compared with the NC group under normoxia and HR conditions (Fig. 4A, B). These results suggested that under HR condition mitochondrial fusion and FUNDC1-mediated mitophagy would be suppressed, and over-expression of miR-130a could increase the suppression effect. Moreover, suppression of miR-130a could promote mitochondrial fusion and FUNDC1-mediated mitophagy, which reversed the myocardial HRI.

A shift in the mitochondrial dynamics balance leads to changes in mitochondrial structural features [28]. To further confirm miR-130a could affect MQC, we analyzed mitochondrial morphology by fluorescence imaging and electron microscopy (EM). As shown in Fig. 4C & E & D, small fragmentary mitochondria would generate under HR condition and mitochondrial swelling. Similarly, over-expression of miR-130a would generate small fragmentary mitochondria and mitochondrial swelling. Meanwhile, suppression of miR-130a would generate a network of long tubular mitochondria (Fig. 4C & E**)** which are beneficial for cell metabolism. Mitophagy will be activated as the number of damaged mitochondria increases to protect myocardial cells [29]. As shown in Fig. 4D & F, mitophagy was suppressed under the HR condition. Interestingly, mitophagy was suppressed in the miR-130a OE group and was activated in the miR-130a KD group (Fig. 4D & F). Taken together, these results stated above suggested the increase of miR-130a generated damaged mitochondria, and at the same time suppressed mitophagy to accelerate myocardial HRI, and suppression of miR-130a could rescue myocardial HRI.

### GJA1 is the target of miR-130a and is down-regulated in I/R rats

**Fig. 5:**
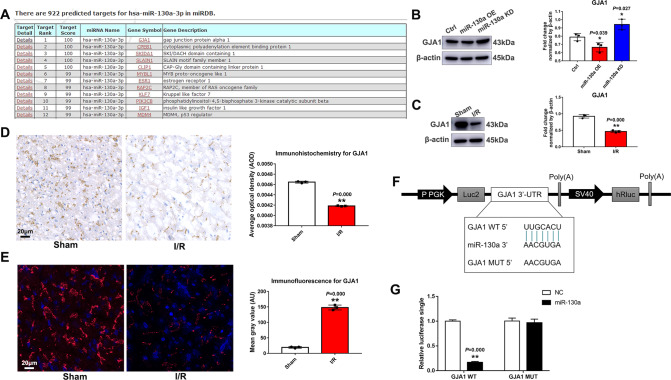
GJA1 is the target of miR-130a and is downregulated in I/R rats. **A** Hsa-miR-130a-3p targets were predicted in the miRDB database. **B** The levels of GJA1 expression were assessed by western blot of Ctrl, miR-130a KD, and miR-130a OE cells. *n* = 3 in each group. Data were expressed as mean ± SD; Statistical significance by two-tailed Student’s t-test, ^*^P < 0.05 *vs*. Ctrl. **C** The levels of GJA1 expression were assessed by western blot in rat myocardial tissues in sham and I/R groups. *n* = 3 in each group. Data were expressed as mean ± SD; Statistical significance by two-tailed Student’s t-test, ^**^P < 0.01 *vs*. the sham group. **D**, **E** GJA1 expression was measured by IHC and IFA. Bar = 20 μm. **F**–**G** MiR-130a targets the GJA1 3’UTR. **F**. Alignment of the GP-miRGLO-GJA1-3’-UTR-WT, GP-miRGLO-GJA1-3’-UTR-Mut luciferase reporter plasmids, and miR-130a sequence. **G**. Luciferase reporter activities of the cells transfected with the luciferase reporter plasmids containing the miR-130a binding sites of GJA1 3’-UTR (GJA1 WT) or not (GJA1 MUT) in the NC and miR-130a mimic groups. Data were expressed as mean ± SD; ^**^P < 0.01 *vs*. NC.

We have demonstrated the increase of miR-130a aggravated mitochondrial dysfunction in myocardial IRI by suppressing mitophagy. To understand the injury mechanism of miR-130a, we first explored which mitochondria-related genes did miR-130a target. MiRDB database was used to predict the genes miR-130a-3p targeted. As shown in Fig. 5A, we noticed the target score of GJA1 was the highest among 922 predicted target genes. To verify whether miR-130a may target the GJA1 3’UTR and regulate GJA1, GP-miRGLO-GJA1-3’-UTR-WT, and GP-miRGLO-GJA1-3’-UTR-Mut luciferase reporter plasmids were designed (Fig. 5F). Luciferase reporter activities were decreased by miR-130a mimics in the wild type (WT) group but not in the mutant (MUT) group, indicating that miR-130a target GJA1 directly (Fig. 5G). Moreover, GJA1 expression could be affected by miR-130a over-expression or knockdown (Fig. 5B), indicating the regulating correlation between miR-130a and GJA1 protein. Our previous studies showed that miR-130a increased in myocardial IRI, which made us wonder whether the level of GJA1 expression changed accordingly. We measured GJA1 expression in myocardial I/R rats by WB (Fig. 5C), immunohistochemistry (IHC) (Fig. 5D), and immunofluorescence assay (IFA) (Fig. 5E), and show consistent results that GJA1 in the I/R group significantly decreased. Therefore, we reasoned that miR-130a aggravated mitochondrial dysfunction in myocardial IRI by regulating GJA1.

### GJA1 enhanced ATP production rate and oxidative phosphorylation, protected cell viability and mitochondrial membrane potential, and activated mitophagy

**Fig. 6:**
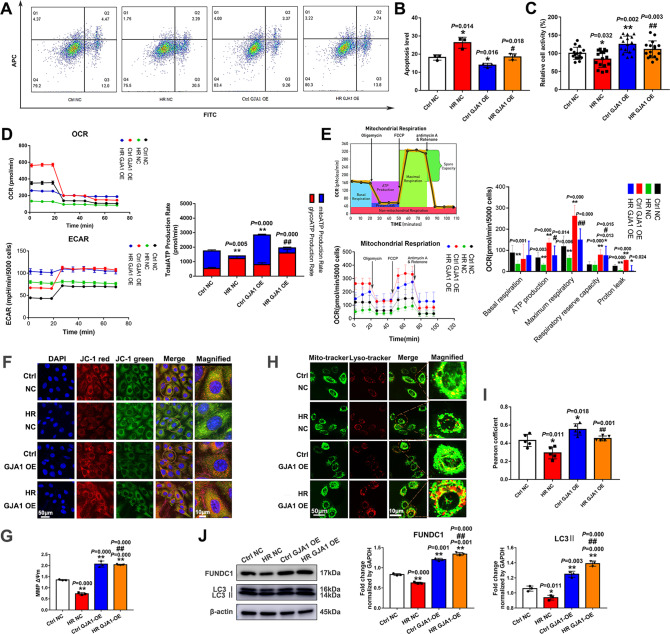
GJA1 protected myocardial cells by decreasing cell apoptosis, enhancing cell viability & ATP production rate & oxidative phosphorylation, protecting mitochondrial membrane potential & activating mitophagy. **A, B** Apoptosis in *H9c2* cells was measured by Flow Cytometry under normoxia or HR conditions with GJA1 OE. *n* = 3 in each group. **C** Cell viability was determined by using a cell counting kit-8 under normoxia or HR conditions with GJA1 OE and absorbance was measured at 450 nm. *n* = 16 in each group. Relative cell activity (%) = [(As-Ab)/(Ac-Ab)] ×100%. As: absorbance of the experimental group; Ab: absorbance of the blank group; Ac: absorbance of the Ctrl NC group. APC: allophycocyanin; FITC: Fluorescein Isothiocyanate. **D** Total ATP production rates were measured by Agilent Seahorse analyzer under normoxia or HR conditions. The ECAR and OCR were measured under basal conditions. By obtaining these data under basal conditions and after serial addition of Rotenone/antimycin A, total cellular ATP Production Rates could be measured in real-time. *n* = 10 in each group. The results of seahorse tests were normalized to the value per 5000 cells. **E** Mitochondrial respiration was measured including ATP production, maximal respiration, Proton leak, spare respiratory capacity, and basal respiration by Agilent Seahorse analyzer under normoxia or HR conditions. *n* = 10 in each group. The results of seahorse tests were normalized to the value per 5000 cells. FCCP: Carbonyl cyanide-4 (trifluoromethoxy) phenylhydrazone. **F–G** MMP was observed with JC-1 staining. Nuclei were stained with DAPI. The cells were observed by laser confocal microscopy. Fluorescence intensity was measured by ImageJ software. Scale bars, 50 μm and 10 μm in magnified. *n* = 3 in each group. **H** The co-localization experiments to observe mitophagy activity. Cells were stained with mito-tracker (green) and lyso-tracker (red). Scale bars, 50 μm and 10 μm in magnified. **I** The quantitative analysis of the co-localization of lysosome and mitochondria was assessed by the Pearson’s correlation coefficient. **J** The levels of FUNDC1-mediated mitophagy-associated proteins were assessed by Western blot. *n* = 3 in each group. Data were expressed as mean ± SD; Statistical significance by two-tailed Student’s t-test, ^*^P < 0.05, ^**^P < 0.01 vs. Ctrl NC; ^*#*^*P* < 0.05, ^##^*P* < 0.01 vs. HR NC.

Based on the previous results, we reasoned that miR-130a aggravated mitochondrial dysfunction in myocardial IRI by regulating GJA1. To test this assumption, initially, we detected the protection of GJA1 on the cell viability and mitochondria through apoptosis, CCK8, ATP production rate, mitochondrial respiration, MMP, and mitophagy. The over-expression of GJA1 could increase the relative cell activity (Fig. 6C) and decrease the apoptosis of *H9c2* cells (Fig. 6A, B) under normoxia and HR conditions. The Over-expression of GJA1 could protect mitochondria in both the Ctrl (normoxia) and HR groups, according to the increase of mitochondrial ATP production rate (Fig. 6D**)**, mitochondrial respiration (Fig. 6E), MMP (Fig. 6F, G). To establish the role of GJA1 on mitophagy, we performed WB to assess the levels of FUNDC1-mediated mitophagy-associated proteins. It was evident that HR injury decreased FUNDC1 and LC3II, however, over-expression of GJA1 significantly increased FUNDC1 and LC3II levels (Fig. 6J). Further, we performed co-localization experiments to observe mitophagy activity where we found a significant decrease in co-localization of lysosomal and mitochondrial structures under HRI (Fig. 6H, I). However, GJA1 over-expression rescued this defect. Hence, these results indicated that GJA1 could rescue the cells under HRI by enhancing ATP production rate and oxidative phosphorylation, protecting MMP, and activating mitophagy.

### MiR-130a regulated mitophagy through GJA1

**Fig. 7:**
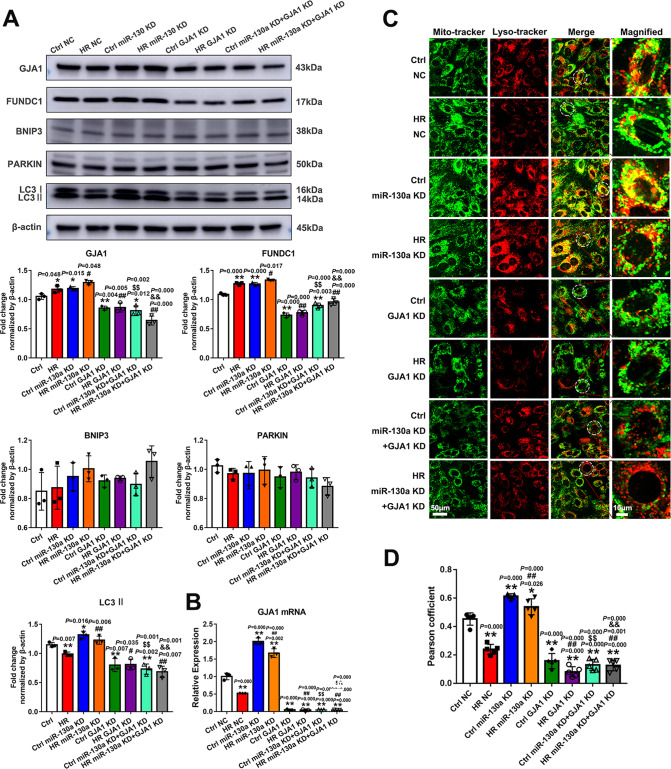
GJA1 was required for miR-130a to aggravate mitochondrial dysfunction in myocardial HRI. **A** The levels of GJA1, FUNDC1, PARKIN, BNIP3, and LC3 were measured by Western blot and quantified. *n* = 3 in each group. **B** The mRNA level of GJA1 expression was detected by qRT-PCR. *n* = 3 in each group. **C** The co-localization experiments were performed to observe mitophagy activity. Cells were stained with Mito-tracker (green) and Lyso-tracker (red). Scale bars, 50 μm and 10 μm in magnified. **D** The quantitative analysis of the co-localization of lysosome and mitochondria was assessed by the Pearson’s correlation coefficient. Data were expressed as mean ± SD; Statistical significance by two-tailed Student’s t-test, ^*^P < 0.05, ^**^P < 0.01 vs. Ctrl NC; ^*#*^*P* < 0.05, ^##^*P* < 0.01 vs. HR NC; ^$$^*P* < 0.01 vs. Ctrl miR-130a KD; ^&&^*P* < 0.01 vs. HR miR-130a KD.

We demonstrated that both miR-130a and GJA1 could regulate FUNDC1-mediated mitophagy, and miR-130a targeted the mitochondria-related gene GJA1. We further wanted to understand the potential effect of miR-130a on the mitochondrial functions of GJA1. Hence, we silenced GJA1 in the miR-130a KD cells under normoxia and HR conditions. Firstly, the mRNA and protein levels of GJA1 expression were detected. Both mRNA and protein levels of GJA1 in the miR-130a KD group significantly increased, and the knockdown of GJA1 could reverse the impact of miR-130a knockdown on GJA1 levels (Fig. 7A, B) under normoxia and HR conditions. Secondly, to identify the association between miR-130a and GJA1 on mitophagy, we performed WB to assess the levels of FUNDC1-mediated mitophagy-associated proteins. As shown in Fig. 7A, the levels of LC3 II and FUNDC1 expression significantly increased in the miR-130a KD group, however, the knockdown of GJA1 in the miR-130a KD group could reverse the effect under Ctrl (normoxia) and HR conditions. Further, we performed co-localization experiments to observe mitophagy activity where we found a significant increase in co-localization of lysosomal and mitochondrial structures in the miR-130a KD group under Ctrl (normoxia) and HR conditions (Fig. 7C, D). However, the knockdown of GJA1 rescued this defect (Fig. 7C, D). Therefore, these results suggest that GJA1 was required for miR-130a to aggravate mitochondrial dysfunction in myocardial HRI by regulating FUNDC1-mediated mitophagy.

## Discussion

Myocardial IRI often occurs during reperfusion therapy of AMI [30]. For patients with AMI, interventional therapy and coronary artery bypass grafting are the main clinical treatment methods [31]. Reperfusion therapy can clear blocked coronary arteries and restore blood flow to the myocardium, nevertheless, it might result in myocardial IRI. Therefore, the diagnosis and treatment of myocardial IRI is an important way in the management of AMI. In the current study, we found the level of miR-130a was significantly increased in the AMI patients. Interestingly, the increase of miR-130a could suppress mitochondrial fusion and FUNDC1-mediated mitophagy to accelerate myocardial HRI. Furthermore, we identified that miR-130a regulated FUNDC1-mediated mitophagy to aggravate mitochondrial dysfunction by targeting GJA1.

Several studies have documented that miR-130a is highly expressed in the heart and plays an important role in the development of cardiovascular diseases [12, 13, 15, 32]. MiR-130a has a protective effect on cardiovascular diseases, for instance, Feng et al. found that miR-130a attenuated cardiac fibrosis after myocardial infarction through transforming growth factor (TGF)-β/Smad signaling by directly targeting TGF-β receptor 1 [33]. Also, there are some studies to find miR-130a might exert a damaging impact on rat cardiomyocytes, for example, down-regulation of miR-130 expression was noted to enhance cardiac protection through PPAR-γ by inhibiting inflammation and myocardial fibrosis [34]. Due to its involvement in the regulation of multiple pathways, its complex mechanism of action remains controversial. Herein, we found miR-130a was significantly increased in the patients with AMI. Recent studies have demonstrated that miRNAs and mitochondria are critically involved in cardiac pathophysiology [35]. Restoring and improving mitochondrial function in cardiomyocytes is an important therapeutic target for delaying the occurrence and development of cardiovascular diseases [36, 37]. Mitochondria are the key triggers cause of cardiac I/R injury [16] because cardiomyocytes contain lots of mitochondria that supply more than 90% of the energy of cardiomyocytes [38] and can promote cardiomyocyte death by inducing apoptosis or necrosis after myocardial reperfusion. Therefore, we attempted to investigate whether the changes in miR-130a expression in hearts had an impact on mitochondrial function. In this study, we found that miR-130a KD could improve mitochondrial function, including an increase in ATP production rate and mitochondrial respiration, a decrease in ROS production, and rescue of MMP. On the contrary, we observed that miR-130a OE augmented mitochondrial dysfunction, including reduced ATP production rate and mitochondrial respiration, increased ROS production, and decreased MMP. As we know, mitochondria have evolved to generate a variety of quality control systems, which are multi-layered, acting on molecular, organelle, and cellular levels to ensure the normal structure and function of cell homeostasis as well as the mitochondrial network [39]. Studies have shown QMC system including mitochondrial dynamics and mitophagy, is essential for mitochondrial function following injury [27]. After mitochondrial dysfunction in myocardial IRI, how did the mechanisms responsible for the elimination of dysfunctional mitochondria work? To answer the question, we explored whether miR-130a could further affect mitochondrial dynamics and mitophagy. Our data indicated that miR-130a significantly reduced mitochondrial fusion, thereby leading to morphological fragmentation of mitochondria. Moreover, mitophagy appeared to be suppressed, which might contribute to the failure of fragmented mitochondria clearing. Mitophagy prevents the accumulation of abnormal mitochondria, while the suppression of mitophagy could lead to cardiomyocyte dysfunction or even death [40]. Accumulating pieces of evidence have demonstrated that mitophagy is critically involved in the development of mitochondrial dysfunction in various settings [41]. Of note, mitochondria can repair themselves through the mitophagy system, by delivering damaged mitochondrial fragments to lysosomes, to reduce the destruction caused by mitochondrial damage to cells, including reducing oxidative stress of cells and restoring mitochondrial energy metabolism [42]. Herein, we further investigated the effect of miR-130a on the activation of the classical mitophagy pathway, in which only the expression of FUNDC1 was significantly altered. Over-expression and knockdown of miR-130a could respectively down-regulate and up-regulate the FUNDC1-mediated mitophagy. Taken together, we believe that there might be a novel signaling pathway involved in the process, that is, miR-130a directly participates in the modulation of FUNDC1-mediated mitophagy by targeting mitochondrial function-related genes.

To clarify the potential mechanism concerning miR-130a in mitophagy, initially, we predicted the target gene of miR-130a. Then, we chose GJA1 as the research object, for the highest score and the mitochondrial metabolic protection of GJA1 [43]. And, GJA1 expression could be affected by miR-130a over-expression or knockdown, indicating the regulating correlation between miR-130a and GJA1. Emerging evidence has indicated that GJA1/CX43 is the main gap junction component. In addition to the formation of gap junctions and semi-channels, novel functions of GJA1 have been documented in recent years, such as mitochondrial function and autophagy modulation [15, 16, 31]. A study by Boengler et al. revealed that GJA1 played an important role in the preservation of mitochondrial function by regulating ROS content and ATP production [22]. In this study, we further noticed that GJA1 could markedly enhance ATP production rate and oxidative phosphorylation, meanwhile could protect the viability and the MMP of rat cardiomyocytes in HRI. It was reported that GJA1 maintained cellular connectivity and homeostasis by regulating autophagy [44]. Martins-Marques et al. found that GJA1 was degraded in the setting of I/R, and it was correlated to autophagic activation [26]. Mitophagy is a form of macro autophagy. Given the regulatory role of GJA1 in autophagy and mitochondrial function, whether GJA1 could modulate mitophagy is worthy of further investigation. Our results that GJA1 could activate FUNDC1-mediated mitophagy might deepen the understanding of the mechanism underlying GJA1-associated mitophagy in cardiomyocytes.

Given the question of whether the regulation of mitophagy by miR-130a was implemented through GJA1, we found that the activation of FUNDC1-mediated mitophagy was markedly reversed after the knockdown of GJA1 in miR-130a KD cells. These results indicated that miR-130a suppressed the activation of FUNDC1-mediated mitophagy via GJA1. The mitochondrial-protein-specific proteases BNIP3, FUNDC1, and Parkin in the QMC are essential receptors in mediating the activity of mitophagy. FUNDC1 is the only upstream regulator found to moderately activate autophagy for mitochondrial protection. A recent report suggested that FUNDC1-induced mitophagy exerted a protective effect on IRI [45]. Mitophagy receptor-FUNDC1 located in the mitochondrial outer membrane can bind to the interaction domain of the key autophagy protein LC3 to activate mitophagy and plays an important role in hypoxia-regulated mitophagy [46]. Under normal conditions, FUNDC1 is phosphorylated by the tyrosine kinase and thus has a low affinity for LC3 [47]. Upon hypoxia, the inactivation of tyrosine kinase increases affinity between FUNDC1 and LC3 and further induces mitophagy [47]. In this study, GJA1 was noted to activate FUNDC1-mediated mitophagy. We reasoned that GJA1 might catalyze the binding of FUNDC1 to LC3 and activate mitophagy by augmenting FUNDC1 dephosphorylation. Therefore, our research on the regulation of FUNDC1-mediated mitophagy might fill the blank on the upstream regulatory signal of FUNDC1 phosphorylation in IRI.

This study must be interpreted in the context of certain limitations. First, we have only studied miR-130a regulated FUNDC1-mediated mitophagy to aggravate mitochondrial dysfunction by targeting GJA1 in *vitro*; however, further research is needed to verify this effect in *vivo*, which will be performed in our ongoing project. Second, the GJA1-FUNDC1 pathway is potentially signaling in mediating the dysfunction of cardiomyocytes, and its interaction network regarding mitophagy needs to be clarified. Third, we have studied the impact of miR-130a on myocardial IRI-related mitophagy and its underlying mechanism, nonetheless, further investigation is required to definite whether miR-130a has clinical significance in myocardial IRI.

**Fig. 8:**
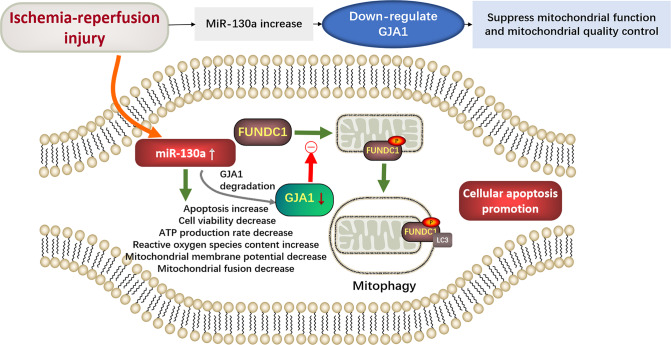
The mechanism of miR-130a aggravating mitochondrial dysfunction in myocardial IRI. In myocardial IRI, miR-130a is up-regulated and the increase of miR-130a can aggravate mitochondrial dysfunction by suppressing mitochondrial function and mitochondrial quality control system, which are represented by the cell viability, ATP production, MMP, and mitochondrial fusion decrease, and the cell apoptosis and ROS content increase. MiR-130a appears to be critically involved in FUNDC1-mediated mitophagy by down-regulating the mitochondria-related gene GJA1. In addition, the reduction of GJA1 might suppress the binding of FUNDC1 to LC3 by reducing FUNDC1 dephosphorylation, and further suppress mitophagy. Therefore, the depression of the FUNDC1-mediated mitophagy contributes to the accumulation of damaged mitochondria and the promotion of cellular apoptosis.

In summary, our data suggest that miR-130a regulates the activation of FUNDC1-mediated mitophagy by targeting GJA1. MiR-130a involves in myocardial IRI by down-regulating GJA1 and suppressing FUNDC1-related mitophagy (Fig. 8). MiR-130a and the GJA1-FUNDC1-associated mitophagy pathway might be potential targets in new-drug development for myocardial IRI, thereby providing a novel therapeutic strategy to combat AMI.

## Materials and methods

### Patient population

Blood samples of AMI patients were collected from the Cardiology Department of the Chinese PLA General Hospital (*n* = 4), Beijing, China. For our study, the diagnosis of AMI was made according to the inclusion criteria from the World Health Organization definition of myocardial infarction: 2008–2009 revision [48]. Informed consent for participation in the study has been obtained, and this consent was written. The study was approved by the Ethical Committee for Medical Research of Chinese PLA General Hospital, Beijing, China.

### Cell culture

The *H9c2* rat cardiomyoblast cell line was obtained from the Qianzhao Xinye Biology Science and Technology Co., Ltd, Beijing, China. The *H9c2* cells were recently authenticated by STR profiling and were tested free of mycoplasma contamination. The *H9c2* cells were grown in complete medium with 10% fetal bovine serum (FBS) (Invitrogen, 10100147, Carlsbad, CA), 1% penicillin-streptomycin, and DMEM high sugar medium (Invitrogen, 11965092, Carlsbad, CA) under 20% O_2_ and 5% CO_2_ at 37 °C.

### Rat myocardial I/R models construction and hypoxia-reoxygenation (HR) treatment of H9c2 cells

The animals and cell lines used in this study were fully compliant with the Guide for the Use of Laboratory Animals published by the US National Institutes of Health. The animal experiment procedure was approved by the Experimental Animal Ethics Committee of the Chinese PLA General Hospital, Beijing, China. Sprague-Dawley (SD) rats (male, 10 weeks, *n* = 12) were purchased from SPF (Beijing) Biotechnology Co., Ltd. The rats were narcotized with 1% Amytal sodium (6 ml/kg), and then were intubated and ventilated by the animal ventilator (Kent Scientific Corporation, S-MVG-RT, Torrington, WY). After a left thoracotomy, the proximal left anterior descending (LAD) coronary artery was ligated with a 5.0 Prolene suture to induce myocardial ischemia. Ischemia was performed for 45 min followed by reperfusion for 6 h, and the heart samples were obtained for experiments. After reperfusion, cardiac parameters were measured by small animal color ultrasound (Fujifilm Visualsonics, Inc., Vevo3100, Bothell, WA) including left ventricular ejection fraction (LVEF), fractional shortening (FS), left ventricular posterior wall; diastolic (LVPW; d), left ventricular posterior wall; systolic (LVPW; s), and heart rate (HR). The sham group underwent the same procedures but did not ligate LAD after threading. The animal sample size was chosen by considering both the specimen availability and experience. In addition, no samples were excluded from the analysis and all rats were randomly assigned to the experiments. There was no blinding method used for the group allocation during experiments. To Simulate IRI in vitro, cells were cultured in Hanks’ balanced salt solution (Invitrogen, 13150016, Carlsbad, CA) under 95% N_2_ and 5% CO_2_ for 45 min, then under normal oxygen conditions while the medium was changed to DMEM re-oxygenating for 6 h.

### Experimental protocol

The grouping and treatment of SD rats and *H9c2* cells in the present study were shown in Fig. 2A.

### Plasmids, siRNA, and inhibitor transfection

For miR-130a OE plasmid construction, it was subcloned rno-miR-130a-3p-sponges into CMV-EGFP-MCS-SV40-neomycin vector. For the knockdown of miR-130a, we used the miR-130a (rat) inhibitor (5’-AUGCCCUUUUAACAUUGCACUG-3’). For GJA1 OE plasmid construction, it was subcloned GJA1 (rat), accession no: NC_051355.1 into pRP[Exp]-NeoR-CMV vector. For the knockdown of GJA1, we used the siRNA_GJA1_ sequences for GJA1 (rat) (Forward: 5’-GCCGCAAUUACAACAAGCATT-3’; Reverse: 5’-UGCUUGUUGUAAUUGCGGCTT-3’). The plasmids, siRNA, and inhibitor were transfected with Lipofectamine^TM^ 3000 Transfection Reagent (Invitrogen, L3000015, Carlsbad, CA), and the cells were collected for subsequent experiments after being cultured for 48 h.

### Databases

The genes targeted by hsa-miR-130a-3p were computationally predicted utilizing the miRDB database (http://www.mirdb.org/).

### Measurement of gene expression by quantificational reverse transcription-polymerase chain reaction (qRT-PCR)

According to the instructions, total RNA was extracted using RNAqueous® Total RNA Isolation Kit (Thermo Fisher Scientific Inc, AM1912, Waltham, MA) and was reverse transcribed using PrimeScript^TM^ RT reagent Kit (Takara Biomedical Technology Co., Ltd., RR047A, Osaka, Japan) for GJA1 analysis. The mRNA level of the GJA1 gene was detected by qRT-PCR using GoTaq qPCR Master Mix Kit (Promega Corporation, A6001, Madison, WI), with β-Actin as a reference gene. For miR-130a analysis, total RNA was reverse transcribed with miRNA ALL-In-One cDNA Synthesis Kit (abm Inc., Zhenjiang, China), and the level of miR-130a was detected using GoTaq qPCR Master Mix Kit, with U6 as an internal control. MiR-130a (rat) specific primer and universal 3’ miRNA reverse primer were purchased from Applied Biological Materials Inc., Richmond, Canada. Other primers were as follows: GJA1 (rat) forward 5′-GCTGGTGGTGTCCTTGGT-3′, reverse 5′-AGTGGAGCCGTTGGTGAG-3′; U6 (rat) forward 5′-CCTGCTTCGGCAGCACA-3′, reverse 5′-AACGCTTCACGAATTTGCGT-3′; β-Actin (rat) forward 5’-CCAACCGTGAAAAGATGACC-3’, reverse 5’-ACCAGAGGCATACAGGGACA-3’.

### Western blot

*H9c2* cells and rat myocardial tissues were lysed with RIPA lysis buffer containing PMSF (Coolaber, SL1020, Beijing, China) for protein extraction. Protein was separated by 10% SDS-PAGE (Bio-Rad Laboratories, Inc., 5671034, Hercules, CA) and transferred to PVDF transfer membranes (Thermo Fisher Scientific Inc, 88520, Waltham, MA). After blocking, the PVDF membranes were incubated overnight with primary antibodies at 4 °C. Then, the PVDF membranes were incubated with a secondary antibody at room temperature for 2 h (1:3000, Abcam, ab6721, Cambridge, England). The images were obtained by the detector (General Electric Company, Imager 600 Amersham Imager 600 series, Boston, MA) using chemiluminescence detection reagents (Applygen Technologies Inc., Beijing, China). Primary antibodies against FUNDC1 (Cell Signaling Technology, 49240, Boston, MA), Parkin (Cell Signaling Technology, 4211, Boston, MA), BNIP3 (Cell Signaling Technology, 12396, Boston, MA), LC3A/B (Cell Signaling Technology, 4108, Boston, MA), GJA1 (Cell Signaling Technology, 83649, Boston, MA), MFN1 (Proteintech Group, Inc., 13798-1-AP, Chicago, IL), MFN2 (Proteintech Group, Inc., 12186-1-AP, Chicago, IL), DRP1 (Abcam, ab184247, Cambridge, England), and β-Actin (Cell Signaling Technology, 4970, Boston, MA) were used in this study, respectively.

### Hematoxylin-eosin (HE) staining and immunohistochemistry (IHC) assay

Myocardial tissues of rats were in sequence dehydrated in gradient, embedded in paraffin, and cut into 5-micrometer slices. After dewaxing, the slices were stained by HE. For the IHC assay, the paraffin slices were dewaxed and treated with 3% hydrogen peroxide at room temperature for 15 min. The slices were boiled in EDTA solution for 3 min and then incubated overnight with GJA1 (Cell Signaling Technology, 83649, Boston, MA) antibody at 4 °C after blocking. The slices were incubated with the second antibody at 37 °C for 20 min, then stained the nucleus. The images were obtained under a microscope (Nikon Instruments Co., Ltd., Ts2, Shanghai, China). Optical density was measured by ImageJ software.

### Immunofluorescence

Myocardial tissues of rats were in sequence dehydrated in gradient, embedded in paraffin, and cut into 5-micrometer slices. For the Immunofluorescence assay, the paraffin slices were dewaxed and treated with 3% hydrogen peroxide at room temperature for 30 min. The slices were blocked with 3% albumin from bovine serum (BSA) for 30 min. Then incubated overnight with GJA1 (1:500, Cell Signaling Technology, 83649, Boston, MA) antibody at 4 °C after blocking. The slices were incubated with the second antibody (1:200, Abcam, ab150083, Cambridge, England) at 37 °C for 30 min, then stained the nucleus. The images were obtained under a microscope (Nikon Instruments Co., Ltd., Ts2, Shanghai, China). Fluorescence intensity was measured by ImageJ software.

### Luciferase Assay

GP-miRGLO-GJA1-3’-UTR-WT and GP-miRGLO-GJA1-3’-UTR-Mut luciferase reporter plasmids were obtained from GenePharma (Shanghai, China). 293 T cells were planted in a 6-well cell culture plate (7.5×10^4^/well), and after 24 h were co-transfected with luciferase reporter plasmids (2 μg) and miR-130a mimics (2 μg) with Lipofectamine^TM^ 3000 Transfection Reagent (Invitrogen, L3000015, Carlsbad, CA). Luciferase assay was performed using the Dual-Luciferase Reporter System (Promega, E1910, Madison, WI). Firefly and Renilla signals were measured using a chemiluminescent enzyme-labeled instrument (Molecular Devices, SpectraMax L, Sunnyvale, CA), and Firefly/Renillia were used to analyze luciferase activity.

### Seahorse real-time ATP rate assay

The Agilent Seahorse XF Real-time ATP Rate Assay (Agilent Technologies, Inc., 103592-100, Santa Clara, CA) was designed to measure living cells’ total ATP production rate. After serial addition of mitochondrial inhibitors (oligomycin and rotenone/antimycin A, Rote/AA), Seahorse Analyzers directly measure real-time extracellular acidification rate (ECAR) and oxygen consumption rate (OCR) of cells-indicators of the two major energy-producing pathways: glycolysis and oxidative phosphorylation. After analysis, cells of each well were counted, and the results were normalized to the value per 5000 cells. The calculation of the mitochondrial and glycolytic ATP production rates provides a real-time measurement of cellular ATP production rates and a quantitative phenotype of cellular energy poise.

### Seahorse mito stress assay

The Agilent Seahorse Cell Mito Stress Assay (Agilent Technologies, Inc., 103015-100, Santa Clara, CA) measures oxidative phosphorylation by directly measuring the OCR of cells. The compounds (oligomycin, FCCP, and a mix of Rote/AA) were serially injected to measure ATP production, maximal respiration, and nonmitochondrial respiration, respectively. Proton leak and spare respiratory capacity are then calculated using these parameters and basal respiration. After analysis, cells of each well were counted, and the results were normalized to the value per 5000 cells.

### Apoptosis detection for live cells

Apoptosis detection for *H9c2* cells was measured using flow cytometry (SONY, SH800, Tokyo, Japan). 10^5^ Cells/group were harvested and stained with Caspase-3 substrate (green fluorescence, Ex/Em = 500/530 nm) and Annexin V-mCherry (red fluorescence, Ex/Em = 587/610 nm) (Beyotime Biotechnology, C1077S, Shanghai, China) for 30 min at 25 °C. Then flow cytometry was performed for apoptosis detection.

### Cell counting kit 8 (CCK8) cell viability assay

The procedure was performed according to the instructions of the Cell Counting Kit-8 (Beyotime Biotechnology, C0038, Shanghai, China), which is widely used for the detection of cell proliferation and cytotoxicity. Briefly, *H9c2* cells were seeded in 96-well plates at a concentration of 5×10^4^ per well and were incubated for 48 h. 10 μL of CCK-8 reagent was added to each well, and the absorbance was measured at 450 nm after 2 h of incubation.

### Detection of mitochondrial morphology by laser confocal microscopy

Cells were incubated with mito-tracker red (Beyotime Biotechnology, C1035, Shanghai, China) at 37 °C for 45 min. The images were obtained by a confocal microscope (Olympus Corporation, FV1000, Tokyo, Japan). The mitochondria structures were observed.

### Transmission Electron Microscopy for observing mitophagy

Observation of mitophagy in *H9c2* cells under transmission electron microscopy. After centrifuged (5000 × *g*, 5 min, 4 °C), cells were harvested and resuspended in 2.5% glutaraldehyde, then were fixed overnight at 4 °C. After washed with buffer, the pellets were fixed in 100 mM cacodylate for 2.5 h. After washing with distilled water, the pellets were dehydrated with ethanol. It was embedded in resin at 65 °C for 48 h. Then, the samples were cut into ultrathin sections and l loaded onto 300-mesh copper grids (Plano GmbH, Marburg, Germany), then stained with uranyl acetate and lead citrate. The grids were viewed under transmission electron microscopy (Hitachi, HT7800, Tokyo, Japan) [49].

### Detection of reactive oxygen species (ROS) content and mitochondrial membrane potential (MMP) by laser confocal microscopy

Tetramethylrhodamine Ethyl Ester **(**TMRE) fluorescent dye (BestBio, BB-41053, Shanghai, China) was used to detect MMP in miR-130a OE and KO cells. 5×10^5^ cells were harvested and resuspended with 500 μl TMRE staining solution/mL. Then the cells were incubated for 15 min at 37 °C. Nuclei were stained with DAPI (Abcam, ab104139, Cambridge, England). The cells were observed by laser confocal microscopy (Leica, STELLARIS 8, Weizler, Germany). Fluorescence intensity was measured by ImageJ software.

JC-1 dye (Thermo Fisher Scientific Inc, T3168, Waltham, MA) was used to detect MMP in GJA1 OE cells. JC-1 is a cationic dye that exhibits potential-dependent accumulation in mitochondria, indicated by a fluorescence emission shift from green (~ 525 nm) to red (~ 590 nm). Cells were dyed with JC-1 dye (2 μM in final concentration) and incubated at 37 °C, 5% CO_2_ for 30 min. Nuclei were stained with DAPI (Abcam, ab104139, Cambridge, England). The cells were observed by laser confocal microscopy (Leica, STELLARIS 8, Weizler, Germany). Fluorescence intensity was measured by ImageJ software. Consequently, mitochondrial depolarization is indicated by a decrease in the red/green fluorescence intensity ratio (ΔΨm).

ROS content was detected by Fluorometric Intracellular ROS Kit (Sigma-Aldrich Corporation, MAK145, St. Louis, MO). Cells were harvested and stained for 20 min at 37 °C according to the instructions of the Kit. Nuclei were stained with DAPI (Abcam, ab104139, Cambridge, England). The cells were observed by laser confocal microscopy (Leica, STELLARIS 8, Weizler, Germany). Fluorescence intensity was measured by ImageJ software.

### Determination of mitophagy activity by co-localization experiment

Cells were incubated with mito-tracker green (Beyotime Biotechnology, C1048, Shanghai, China) and lyso-tracker red (Beyotime Biotechnology, C1046, Shanghai, China) at 37 °C for 45 min, respectively. The images were obtained by a confocal microscope (Olympus Corporation, FV1000, Tokyo, Japan). The colocalization of mitochondria and lysosome structures was observed, and the Pearson’s correlation coefficient was used for quantitative analysis of the co-localization of lysosomal and mitochondrial. ImageJ software calculated the Pearson’s correlation coefficient.

### Statistical analysis

Statistical analysis was conducted using GraphPad Prism 7 (Graphpad Software Inc., San Diego, CA). Data were expressed as mean ± standard deviation (SD), each of which represents at least three independent experiments. All data were first tested for normality via the Kolmogorov-Smirnov test and for equal variances via Levene’s test. Comparing continuous data variables among multi-group, we carried out an ordinary one-way ANOVA test and the two-group were analyzed by Student’s t-test. The 95% confidence intervals (CI) for the parameters in our analysis were estimated and a two-sided *P* < 0.05 (the threshold) was defined as being statistically significant.

## Supplementary information

Original western blots

## Data Availability

The datasets used and/or analyzed during the current study are available from the corresponding author upon reasonable request.
