# Supplementary material for: MiR-130a-3p regulates FUNDC1-mediated mitophagy by targeting GJA1 in myocardial ischemia/reperfusion injury
Source: Cell Death Discov. 2023 Feb 25;9:77. doi: 10.1038/s41420-023-01372-7 (PMC9968299; doi:10.1038/s41420-023-01372-7)
Supplement: Supplementary file 1 — Original western blots [file 41420_2023_1372_MOESM1_ESM.pdf]

Western blotting

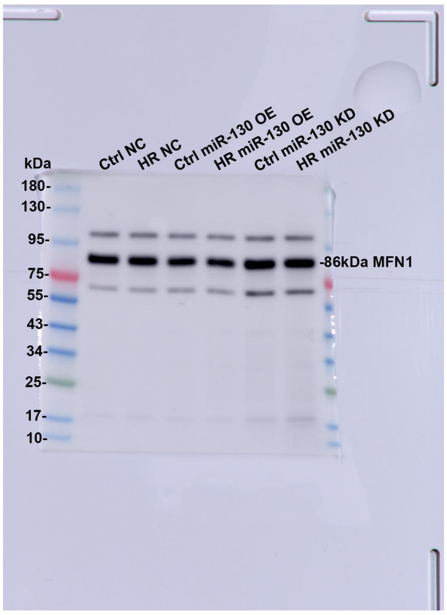

Original Image for Fig. 4A-MFN1

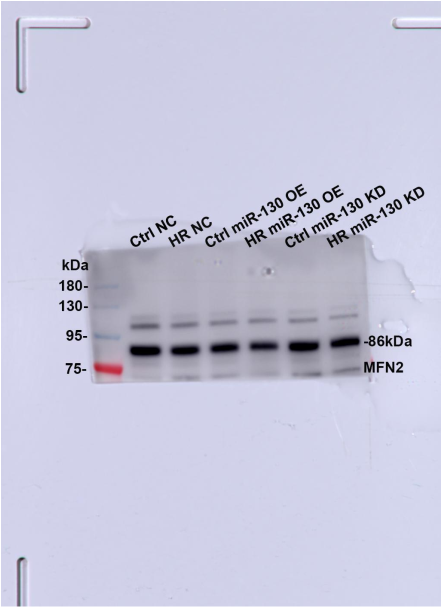

Original Image for Fig. 4A-MFN2

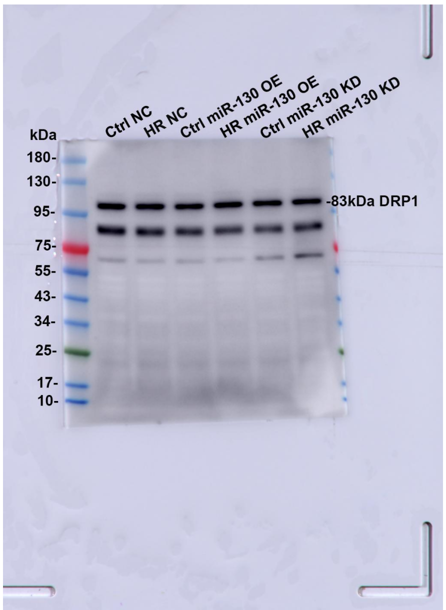

Original Image for Fig. 4A-DRP1

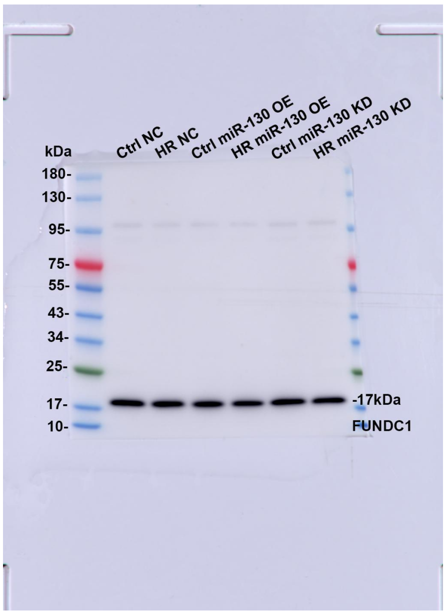

Original Image for Fig. 4A-FUNDC1

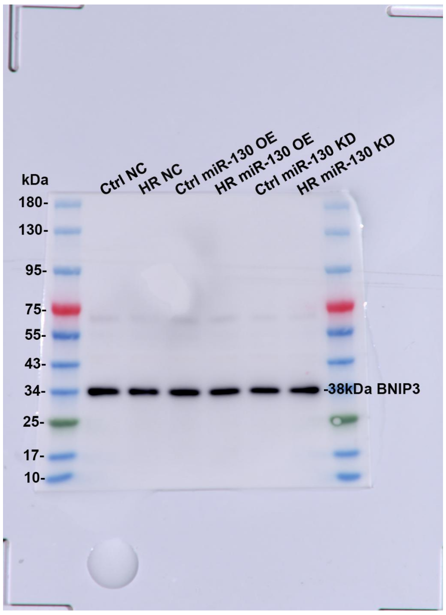

Original Image for Fig. 4A-BNIP3

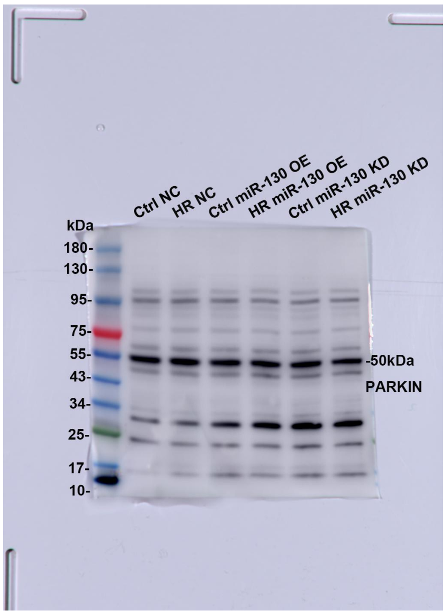

Original Image for Fig. 4A-PARKIN

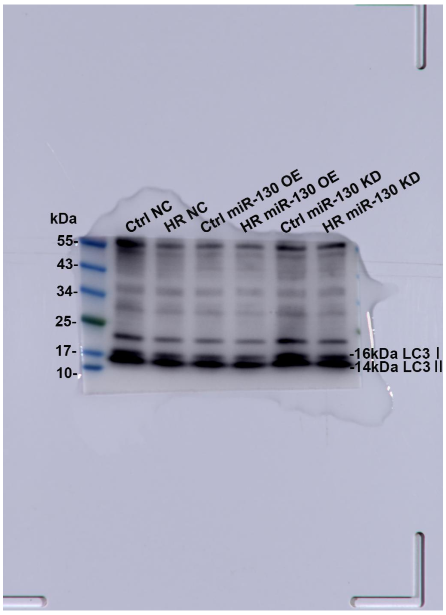

Original Image for Fig. 4A-LC3

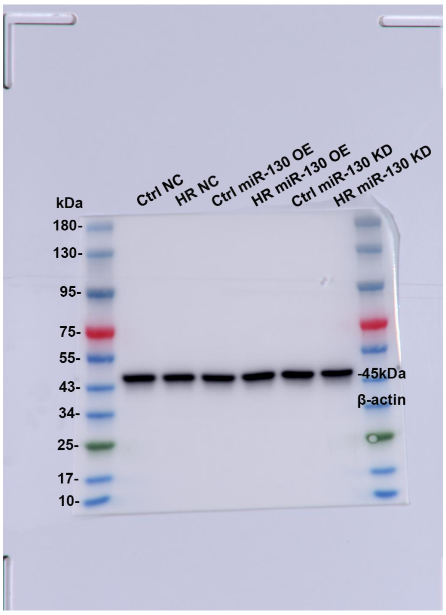

Original Image for Fig. 4A-β-actin

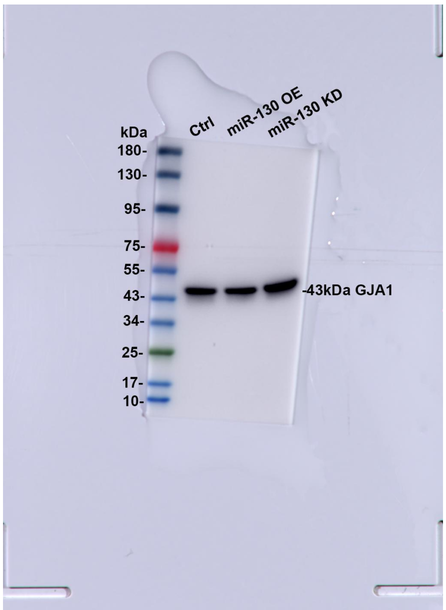

Original Image for Fig. 5B-GJA1

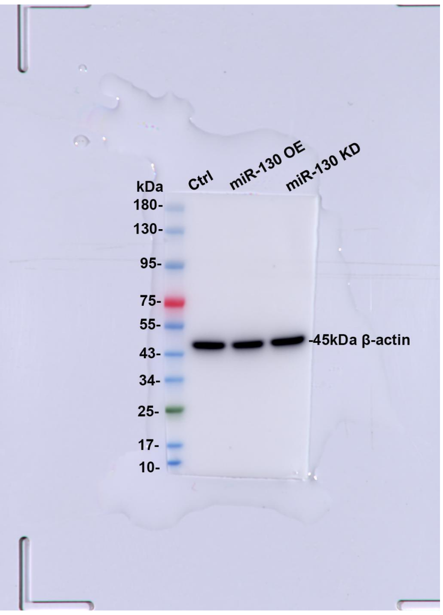

Original Image for Fig. 5B-β-actin

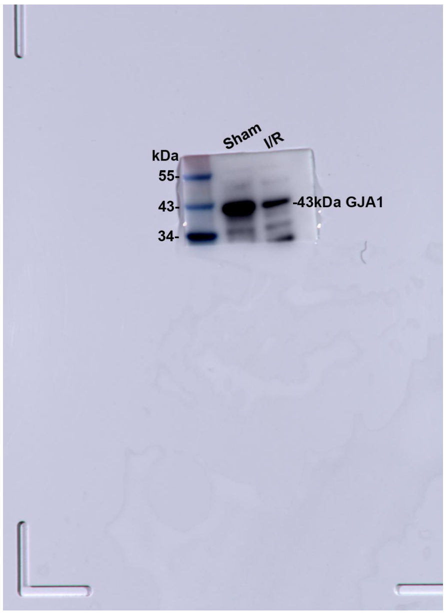

Original Image for Fig. 5C-GJA1

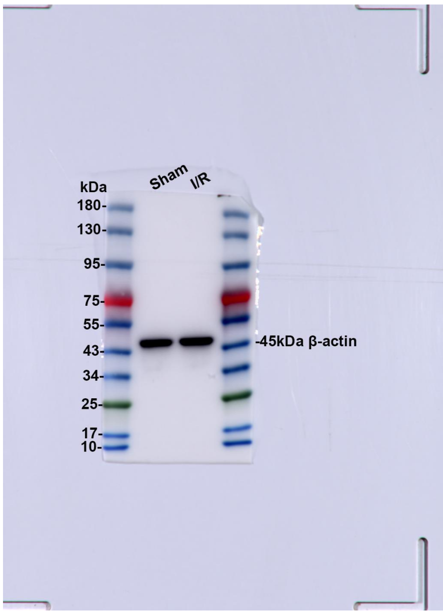

Original Image for Fig. 5C-β-actin

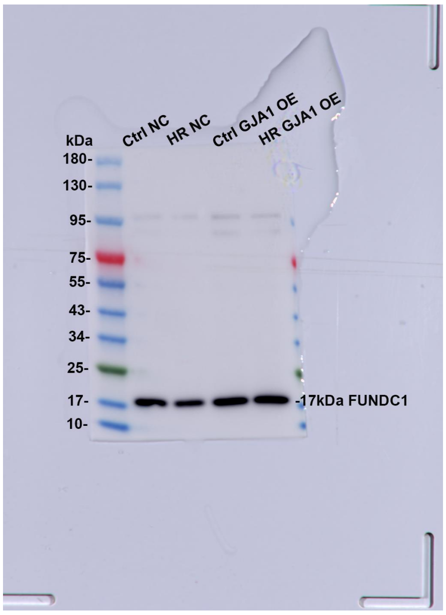

Original Image for Fig. 6J-FUNDC1

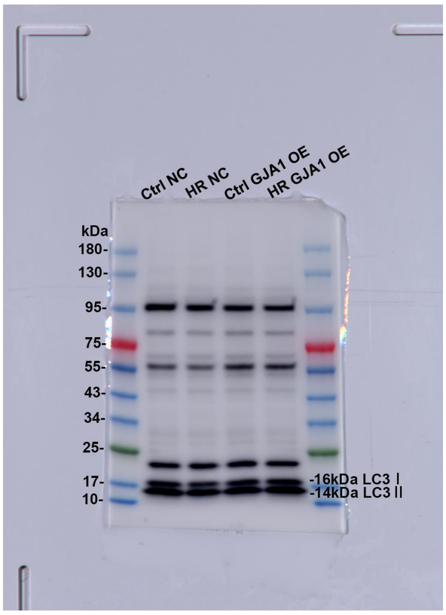

Original Image for Fig. 6J-LC3

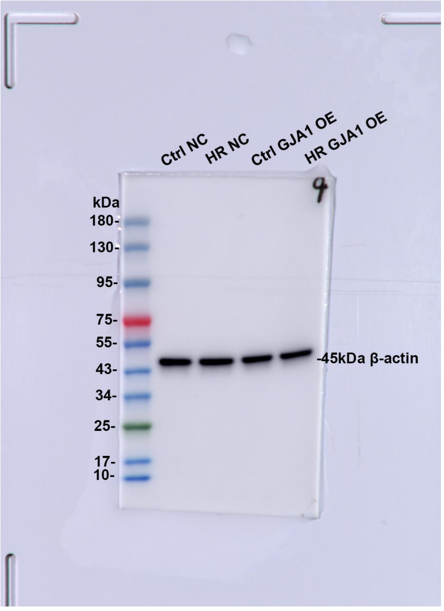

Original Image for Fig. 6J-β-actin

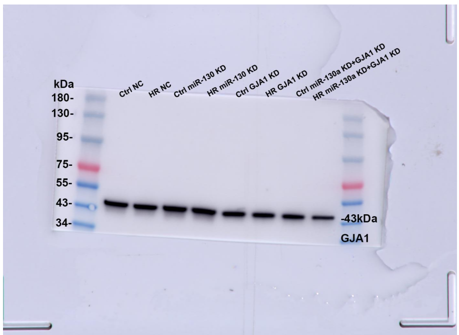

Original Image for Fig. 7A-GJA1

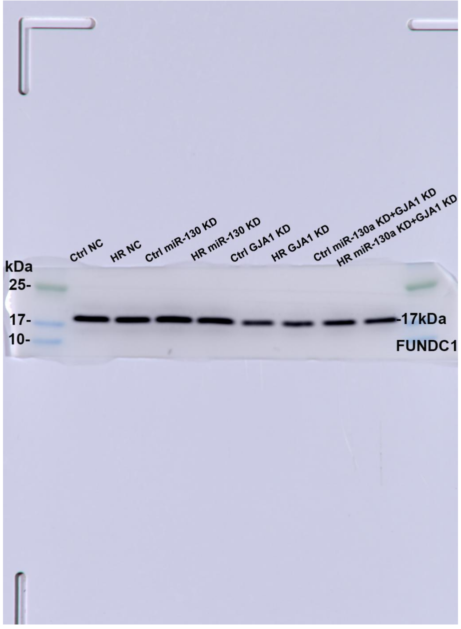

Original Image for Fig. 7A-FUNDC1

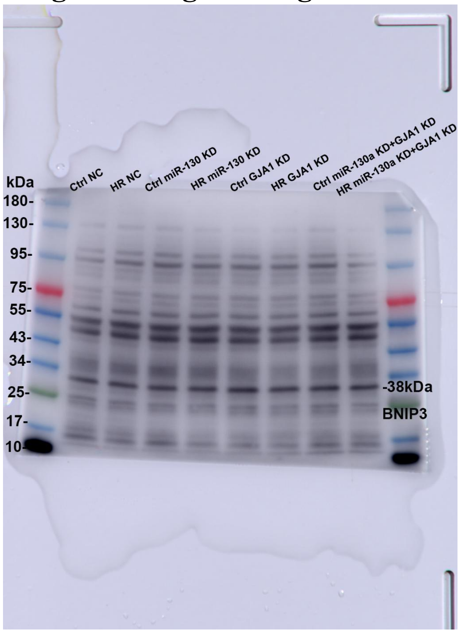

Original Image for Fig. 7A-BNIP3

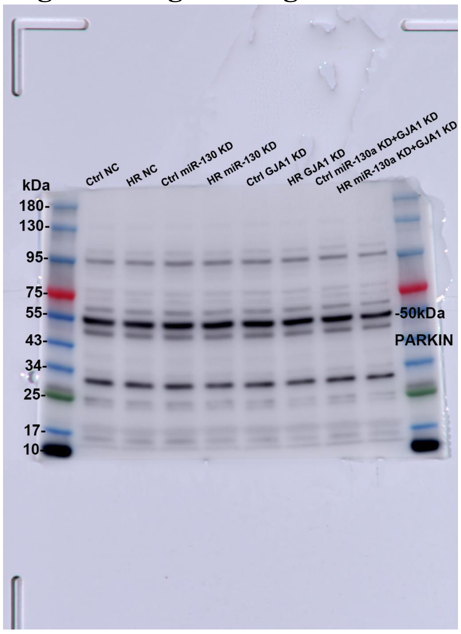

Original Image for Fig. 7A-PARKIN

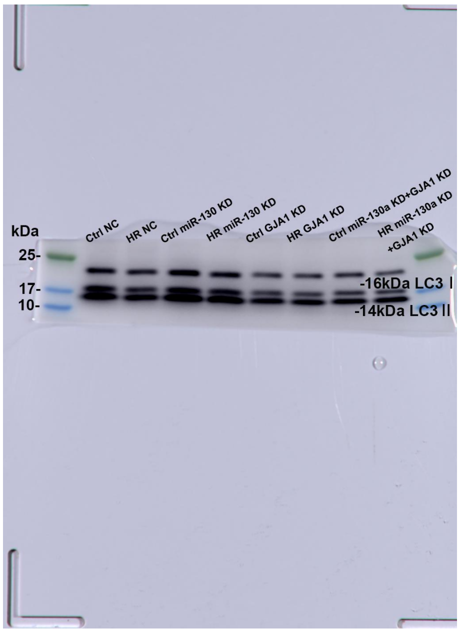

Original Image for Fig. 7A-LC3

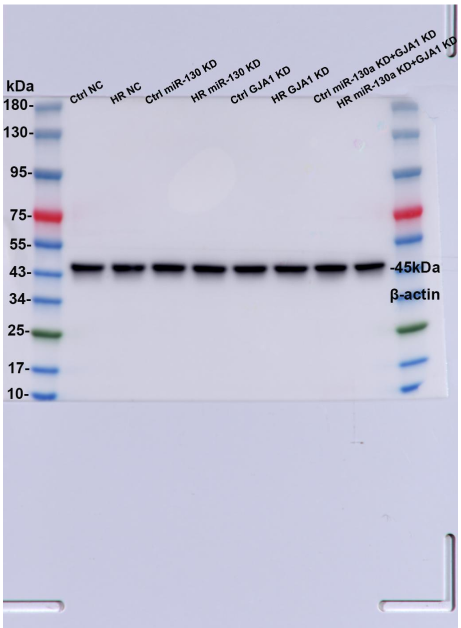

Original Image for Fig. 7A-β-actin
